# Thromboembolic and bleeding risk of periprocedural bridging anticoagulation: A systematic review and meta‐analysis

**DOI:** 10.1002/clc.23336

**Published:** 2020-01-16

**Authors:** Hsien‐Cheng Kuo, Feng‐Lin Liu, Jui‐Tai Chen, Yih‐Giun Cherng, Ka‐Wai Tam, Ying‐Hsuan Tai

**Affiliations:** ^1^ Department of Anesthesiology Shuang Ho Hospital, Taipei Medical University New Taipei City Taiwan; ^2^ Department of Anesthesiology, School of Medicine College of Medicine, Taipei Medical University Taipei Taiwan; ^3^ Division of General Surgery, Department of Surgery Shuang Ho Hospital, Taipei Medical University New Taipei City Taiwan; ^4^ Division of General Surgery, Department of Surgery, School of Medicine College of Medicine, Taipei Medical University Taipei Taiwan; ^5^ Center for Evidence‐Based Health Care Shuang Ho Hospital, Taipei Medical University New Taipei City Taiwan

**Keywords:** anticoagulant, bridging therapy, hemorrhage, thrombosis

## Abstract

The risk and benefit of periprocedural heparin bridging is not completely clarified. We aimed to assess the safety and efficacy of bridging anticoagulation prior to invasive procedures or surgery. Heparin bridging was associated with lower risks of thromboembolism and bleeding compared to non‐bridging. PubMed, Ovid and Elsevier, and Cochrane Library (2000‐2016) were searched for English‐language studies. Studies comparing interrupted anticoagulation with or without bridging and continuous oral anticoagulation in patients at moderate‐to‐high thromboembolic risk before invasive procedures were included. Primary outcomes were thromboembolic events and bleeding events. Mantel‐Haenszel method and random‐effects models were used to analyze the pooled risk ratio (RR) and 95% confidence interval (CI) for thromboembolic and bleeding risks. Eighteen studies (six randomized controlled trials and 12 cohort studies) were included (N = 23 364). There was no difference in thromboembolic risk between bridged and non‐bridged patients (RR: 1.26, 95% CI: 0.61‐2.58; RCTs: RR: 0.71, 95% CI: 0.23‐2.24; cohorts: RR: 1.45, 95% CI: 0.63‐3.37). However, bridging anticoagulation was associated with higher risk of overall bleeding (RR: 2.83, 95% CI: 2.00‐4.01; RCTs: RR: 2.24, 95% CI: 0.99‐5.09; cohorts: RR: 3.09, 95% CI: 2.07‐4.62) and major bleeding (RR: 3.00, 95% CI: 1.78‐5.06; RCTs: RR: 2.48, 95% CI: 1.29‐4.76; cohorts: RR: 3.22, 95% CI: 1.65‐6.32). Bridging anticoagulation was associated with increased bleeding risk compared to non‐bridging. Thromboembolism risk was similar between two strategies. Our results do not support routine use of bridging during anticoagulation interruption.

## 1 INTRODUCTION

An estimated 2.5 million patients use oral anticoagulants for the prevention of arterial thromboembolic events in North America, and one‐tenth of them require temporary interruption in preparation for an elective procedure or surgery.^1^, ^2^ However, the safety and efficacy of bridging anticoagulation is not completely clarified for patients who need an anticoagulation interruption before invasive procedures. Two main concerns remain unsolved, the risk of thromboembolism, and the risk of bleeding.^1^, ^2^

To reduce the bleeding risk for patients undergoing invasive procedures, oral anticoagulant is typically interrupted prior to the procedure, and then continued when hemostasis is achieved postprocedurally.^1^, ^2^ Because the interruption of anticoagulation may expose patients to the risk of thromboembolism, heparin bridging (unfractionated heparin [UFH] or low‐molecular‐weight heparin [LMWH]) is administered to minimize the period of inadequate level of anticoagulation.^1^, ^2^ It is of great importance to confirm whether bridging therapy reduces thromboembolic risk and to ascertain the safety of bridging therapy in relation to bleeding risk.^3^

There have been many published articles related to bridging anticoagulation,^4^, ^5^ but the quality of evidence with best practices is uneven across studies. Current guidelines from the 2019 American College of Cardiology/American Heart Association suggest bridging anticoagulation used in patients with a high thrombosis risk, such as certain mechanical valve prostheses or recent pulmonary embolism during interruption of vitamin K antagonist (VKA) therapy.^3^ However, these recommendations are primarily based on observational studies and experts' opinions.^3^

Although Siegal and colleagues concluded that bridging anticoagulation increases bleeding risk and produces similar thromboembolic risk, their review included only one underpowered randomized trial together with some observational studies with no control arm to assess the safety and efficacy of bridging therapy.^6^ To better clarify the risk and benefit of bridging therapy, we updated the current published data and conducted a meta‐analysis to compare the periprocedural thromboembolic and hemorrhagic risks between patients receiving interrupted anticoagulation with or without bridging therapy and continuous oral anticoagulation.

## 2 MATERIALS AND METHODS

### 2.1 Data sources and searches

We used the Preferred Reporting Items for Systematic Reviews and Meta‐analyses (PRISMA) guidelines^7^ and performed a search of PubMed, Ovid and Elsevier, and Cochrane Library for published randomized and observational studies in English from January 1, 2000 to August 30, 2016. We searched with keywords “long‐term oral anticoagulant,” “chronic oral anticoagulant,” “periprocedural anticoagulant,” “perioperative anticoagulant,” “uninterrupted anticoagulant,” “continued oral anticoagulant,” “interrupted anticoagulant,” “unfractionated heparin bridging,” and “low‐molecular‐weight heparin bridging.” References of articles and previous meta‐analyses were also reviewed to confirm that no studies were missed.

### 2.2 Data retrieval and quality evaluation

Included studies met all of the following criteria: patients ≥18 years of age, long‐term use of oral anticoagulant before a procedure, elective invasive procedure or surgery, comparison of periprocedural bridging anticoagulation and non‐bridging management (continuous oral anticoagulation or interrupted oral anticoagulation without bridging therapy), reporting of both thromboembolic and bleeding events, and articles published in peer‐reviewed journals. Studies with unclear reporting of thromboembolic or bleeding events, case reports, or letters to the editor were excluded. Studies with no control arm or designed specific to patients using novel oral anticoagulants (NOACs) were also excluded. Two authors (H.C.K. and F.L.L.) independently reviewed and collected data, including study design, patient characteristics of interest and relevant outcomes. The third blinded reviewer (Y.H.T.) resolved any disagreements between reviewers.

Patients were classified as bridged if they received any bridging therapy with UFH or LMWH in the periprocedural period. Patients were classified as non‐bridged if they interrupted oral anticoagulation without heparin bridging or continued use of oral anticoagulants during periprocedural period. Classification of high and low thromboembolic risk was based on the definitions described in the primary studies.

Primary outcomes were thromboembolic events and overall bleeding events. Secondary outcome was major bleeding events. Thromboembolic events were defined as stroke, transient ischemic attack, systemic embolism, myocardial infarction, deep vein thrombosis, or pulmonary embolism occurring during the follow‐up period. Major bleeding was defined as a need for transfusion, drop in hemoglobin >2 g/L, requirement for surgical hemostasis, need for rehospitalization, intracranial hemorrhage, or fatal bleeding.

Study quality was assessed with criteria adapted from the Antithrombotic Therapy and Prevention of Thrombosis, ninth ed: American College of Chest Physicians Evidence‐Based Clinical Practice Guidelines.^8^ Disagreements on data acquisition were resolved by consensus with the third reviewer.

### 2.3 Data synthesis and analysis

Data were pooled by using the Mantel‐Haenszel method, and the random‐effects model was performed to generate risk ratios (RRs) by using the RevMan software, version 5.3 (Copenhagen: The Nordic Cochrane Centre, The Cochrane Collaboration; 2014).^9^ The *I* ^2^ statistics was used to check for quantitative heterogeneity of results; it defines low heterogeneity with *I* ^2^ < 25%, moderate heterogeneity with *I* ^2^ between 25% and 50%, and high heterogeneity with *I* ^2^ more than 50%. A funnel plot was used to assess a potential publication bias. Visual estimation was performed for the asymmetry of the funnel plot. Descriptive statistics were generated with the SAS software, version 9.4 (SAS Institute Inc., Cary, North Carolina).

## 3 RESULTS

**Figure 1:**
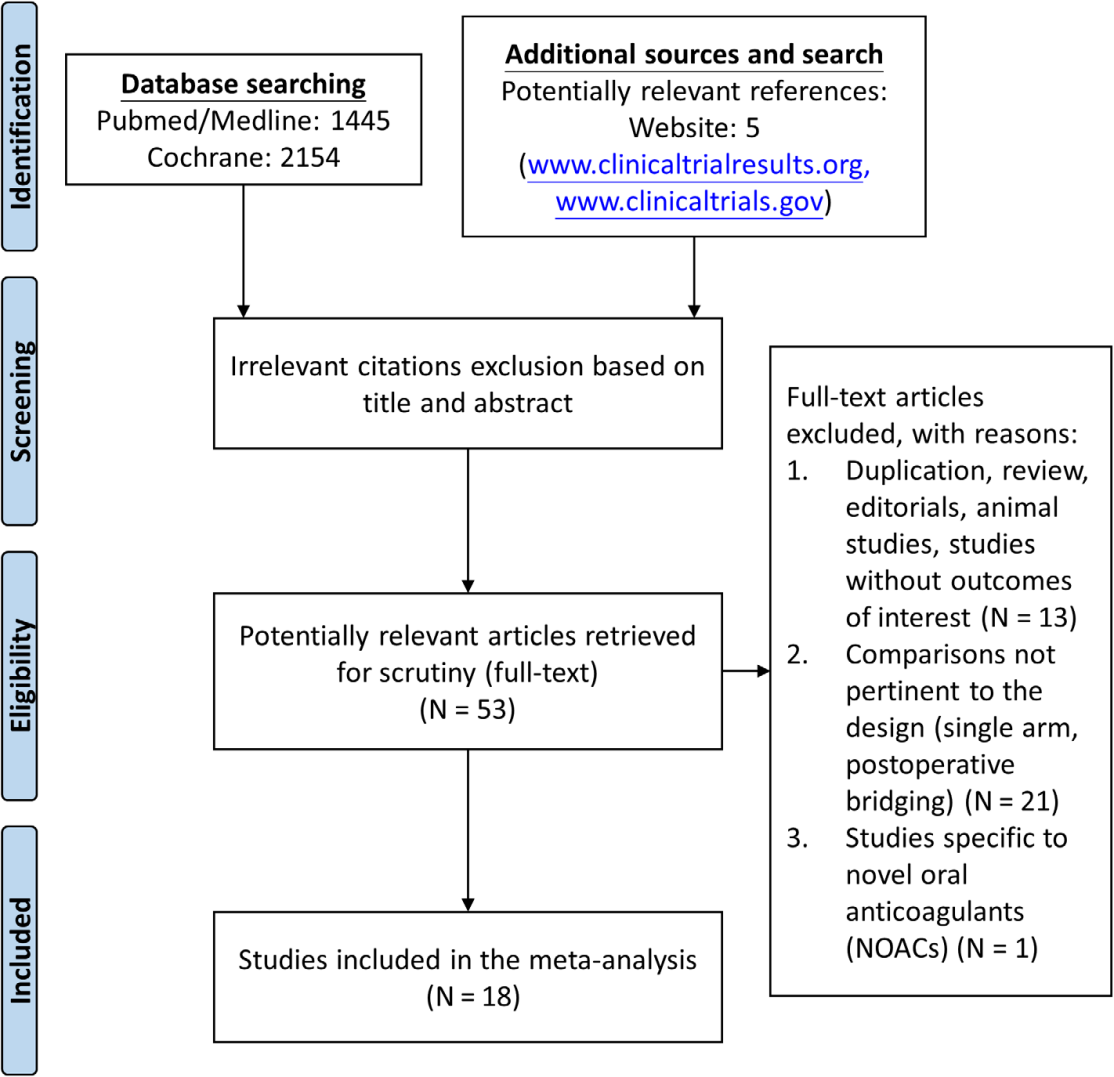
Identification of eligible studies according to the PRISMA statement

The PRISMA flow diagram is described in Figure 1. We identified a total of 18 studies^10^, ^11^, ^12^, ^13^, ^14^, ^15^, ^16^, ^17^, ^18^, ^19^, ^20^, ^21^, ^22^, ^23^, ^24^, ^25^, ^26^, ^27^ (six randomized controlled trials,^12^, ^14^, ^18^, ^20^, ^23^, ^25^ 12 cohort studies^10^, ^11^, ^13^, ^15^, ^16^, ^17^, ^19^, ^21^, ^22^, ^24^, ^26^, ^27^) and enrolled 23 364 patients. Patients were divided into the bridged (N = 5421) and non‐bridged (N = 17 943) groups. Baseline characteristics of the included studies are listed in Table 1. Indications for anticoagulation were reported in all studies, including atrial fibrillation, mechanical heart valve, previous pulmonary embolism, and venous thromboembolism. The types of invasive procedures varied across studies as follows: endoscopic (5 of 18), orthopedic (7 of 18), dental (8 of 18), ophthalmologic (6 of 18), cardiac device implantation (13 of 18), dermatologic (3 of 18), and angiographic (5 of 18). Surgical procedures comprised urologic (5 of 18), general (8 of 18), abdominal (6 of 18), vascular (5 of 18), gynecologic (2 of 18), cardiothoracic (8 of 18), and neurologic (4 of 18). Among the included studies, seven studies used LWMH as the single bridging therapy; two studies used UFH as the single bridging therapy; nine studies used either UFH or LWMH. Besides, there were eight studies including patients with continuous oral anticoagulation^12^, ^13^, ^14^, ^17^, ^18^, ^19^, ^20^, ^23^ and 11 studies comparing bridging anticoagulation with interrupted oral anticoagulant without bridging therapy.^10^, ^11^, ^17^, ^18^, ^19^, ^21^, ^22^, ^24^, ^25^, ^26^, ^27^ Periprocedural bridging strategies are shown in Table S1. Before invasive procedures or surgery, LMWH was discontinued within 24 hours in 19% of studies, beyond 24 hours in 44% of studies, and at unspecified time in 37% of studies. Postprocedurally, LMWH was reinitiated beyond 24 hours in 54% of studies and at unspecified or varied time in 46% of studies. VKA was reinitiated within 24 hours in 56% of studies, beyond 24 hours in 11% of studies and at unspecified or varied time in 33% of studies. Individual studies may have used different strategies of VKA discontinuation period and VKA or LMWH reinitiation time.

**Table 1:** Characteristics of included studies

Author	Design	Indication of VKA	No. of patients	Anticoagulation management	Type of heparin	Follow‐up time
Wysokinski et al, 2008 10	Cohort, prospective	non‐valvular Af	B: 204 I: 182	B: warfarin withheld with bridging management I: warfarin withheld without bridging management	UFH or LMWH	90 days
Garcia et al, 2008 11	Cohort, prospective	Af, venous thromboembolism, mechanical heart valve	B: 108 I: 1185	B: warfarin withheld with bridging management I: warfarin withheld without bridging management	LMWH	30 days
Bajkin et al, 2009 12	RCT	miscellaneous	B: 105 C: 109	B: VKA withheld with bridging management C: VKA continued	LMWH	30 days
Tischenko et al, 2009 13	Cohort, prospective	Af, mechanical heart valve, recent venous thromboembolism	B: 38 C: 117	B: warfarin withheld with bridging management C: warfarin continued	LMWH	30 days
Tolosana et al, 2009 14	RCT	miscellaneous	B: 51 C: 50	B: VKA withheld with bridging management C: VKA continued	UFH	45 days
Daniels et al, 2009 15	Cohort, prospective	mechanical heart valve	B: 342 I + C: 213	B: warfarin withheld with bridging management I + C: warfarin withheld without bridging management or warfarin continued	UFH or LMWH	90 days
McBane et al, 2010 16	Cohort, retrospective	VTE under chronic anticoagulation therapy	B: 514 I + C: 261	B: warfarin withheld with bridging management I + C: warfarin withheld without bridging management or warfarin therapy continued	LMWH	90 days
Ahmed et al, 2010 17	Cohort, retrospective	Af, mechanical heart valve, DVT, left ventricular thrombus	B: 123 I: 114 C: 222	B: warfarin withheld with bridging management I: warfarin withheld without bridging management C: warfarin continued	UFH or LMWH	8 weeks
Cheng et al, 2011 18	RCT	miscellaneous	B: 7 I: 43 C: 50	B: warfarin withheld with bridging management I: warfarin withheld without bridging management C: warfarin continued	UFH or LMWH	4 to 6 weeks
Li et al, 2011 19	Cohort, retrospective	miscellaneous	B: 199 I: 243 C: 324	B: warfarin withheld with bridging management I: warfarin withheld without bridging management C: warfarin continued	UFH or LMWH	30 days
Birnie et al, 2013 20	RCT	miscellaneous (risk of thromboembolism >5%)	B: 325 C: 334	B: warfarin withheld with bridging management C: warfarin continued	UFH or LMWH	1 to 2 weeks
Yokoshiki et al, 2013 21	Cohort, retrospective	Af, left ventricular thrombus, and mechanical heart valve	B: 34 I: 59	B: warfarin withheld with bridging management I: warfarin withheld without bridging management	UFH	30 days
Sherwood et al, 2014 22	Cohort, prospective	non‐valvular Af and ≥ 2 stroke risk factors	B: 483 I: 7072	B: VKA withheld with bridging management I: VKA withheld without bridging management	LMWH	30 days
Schulman et al, 2014 23	RCT	Af, mechanical heart valve, left ventricular thrombus, VTE	B: 85 C: 86	B: warfarin withheld with bridging management C: warfarin continued (reduced dose)	LMWH	30 days
Steinberg et al, 2015 24	Cohort, prospective	Af	B: 514 I: 1766	B: VKA or NOACs withheld with bridging management I: VKA or NOACs withheld without bridging management	UFH or LMWH	30 days
Douketis (A) et al, 2015 25	RCT	non‐valvular Af and at least one of the CHADS2 risk factors	B: 934 I: 950	B: warfarin withheld with bridging management I: warfarin withheld without bridging management	LMWH	30 days
Douketis (B) et al, 2015 26	Cohort, prospective	non‐valvular Af and ≥1 stroke risk factors	B: 800 I: 3306	B: warfarin or NOACs withheld with bridging management I: warfarin or NOACs withheld without bridging management	UFH or LMWH	30 days
Clark et al, 2015 27	Cohort, retrospective	chronic warfarin therapy for secondary prevention of VTE	B: 555 I: 1257	B: warfarin withheld with bridging management I: warfarin withheld without bridging management	UFH or LMWH	30 days

Af, atrial fibrillation; B, bridged group; C, continued group; DVT, deep vein thrombosis; I, interrupted group; LMWH, low molecular weight heparin; NOACs, novel oral anticoagulants; RCT, randomized controlled trial; UFH, unfractionated heparin; VKA, vitamin K antagonist; VTE, venous thromboembolism.

### 3.1 Study quality assessment

Table S2 showed the assessment of study quality: six studies of the 18 included study used a randomized design; among them, three studies conducted a high‐quality randomization with allocation concealment.^14^, ^20^, ^25^ One trial was double‐blind and placebo‐controlled.^25^ Besides, the 12 cohort studies were heterogeneous in the quality of patient enrollment process, blinded assessment of outcome, and reporting of loss to follow‐up.

### 3.2 Risk of thromboembolism

**Figure 2:**
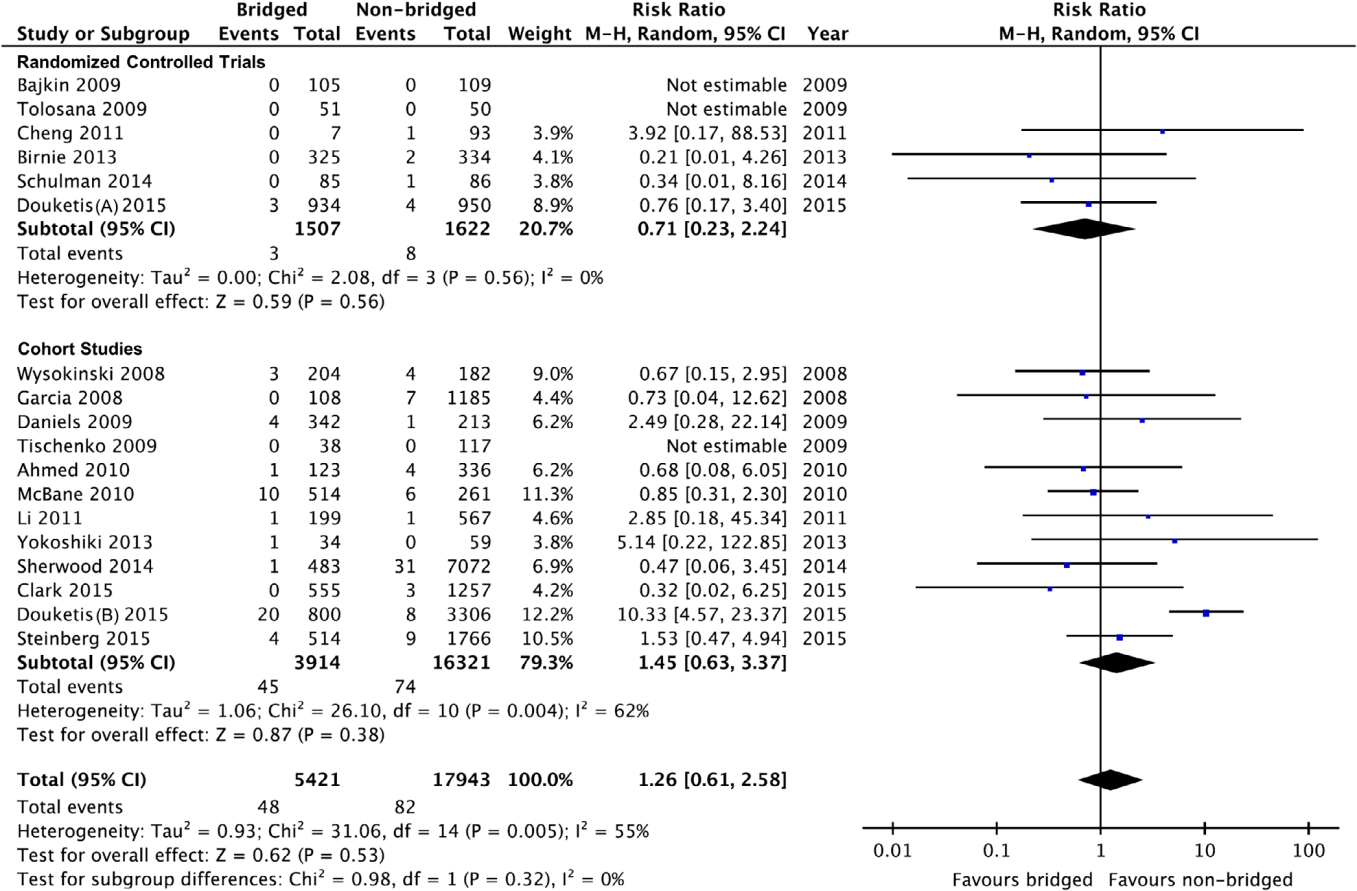
Forest plot of thromboembolic events between bridged and non‐bridged groups. CI, confidence interval; M‐H, Mantel–Haenszel

When data were pooled across studies, there was no difference in the risk of thromboembolism between the bridged and non‐bridged group (48 events of 5421 bridged patients and 82 events of 17 943 non‐bridged patients), RR: 1.26 (95% CI: 0.61‐2.58, *P* = .53; *I* ^2^ = 55%; Figure 2). Funnel plot manufactured for the outcome of thromboembolic events revealed no obvious publication bias.

We performed separate analyses to assess the effect of study designs; nevertheless, there was no difference in the thromboembolic risk between groups in randomized controlled trials (RR: 0.71, 95% CI: 0.23‐2.24, *P* = .56; *I* ^2^ = 0%; Figure 2).

### 3.3 Risk of bleeding

**Figure 3:**
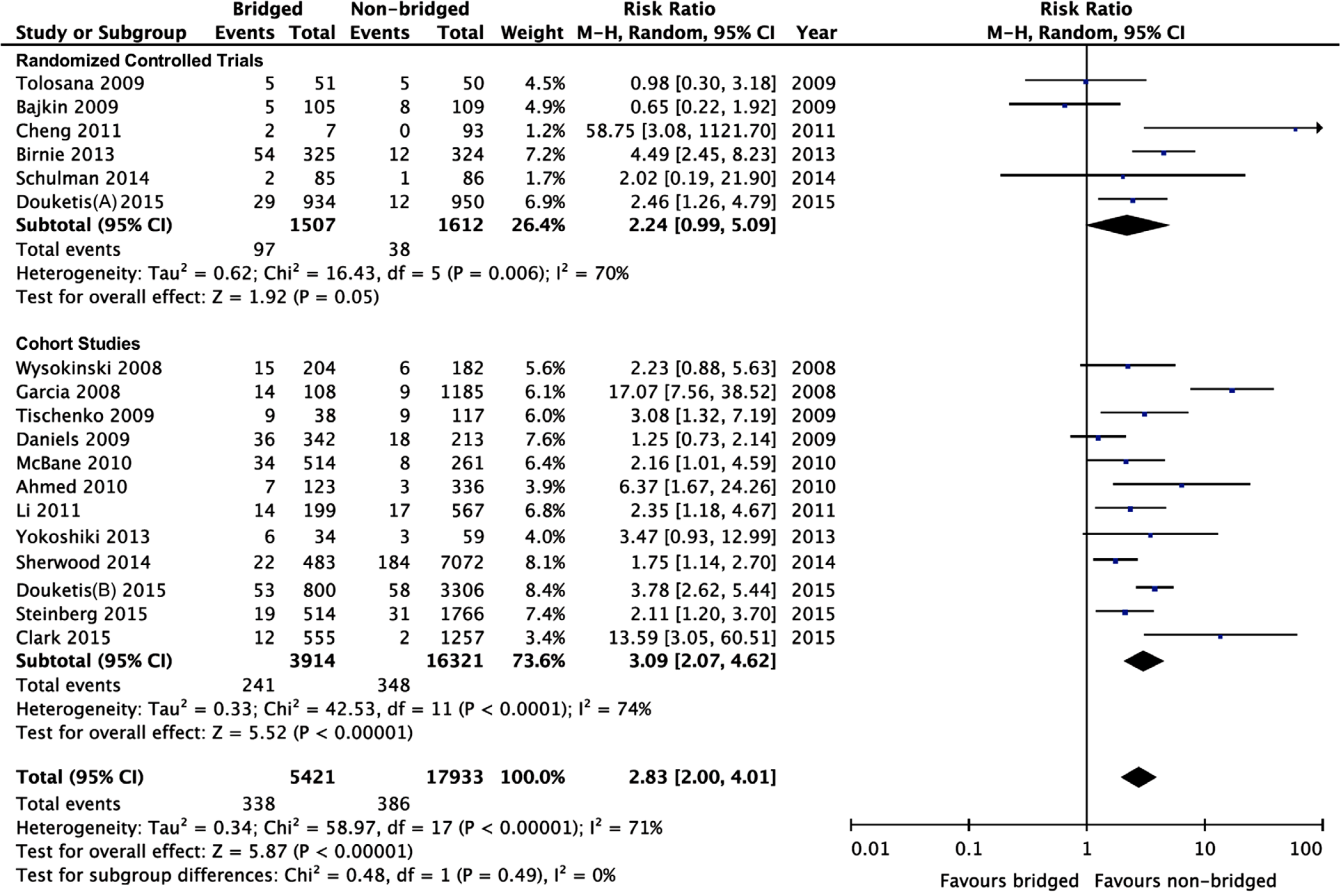
Forest plot of overall bleeding events between bridged and non‐bridged groups. CI, confidence interval; M‐H, Mantel‐Haenszel

The overall bleeding risk in the bridged group was significantly higher compared to non‐bridged group regardless of study design with high heterogeneity of results, RR: 2.83 (95% CI: 2.00‐4.01, *P* < .0001; *I* ^2^ = 71%; Figure 3). Funnel plot manufactured for the outcome of bleeding events showed no obvious publication bias.

**Figure 4:**
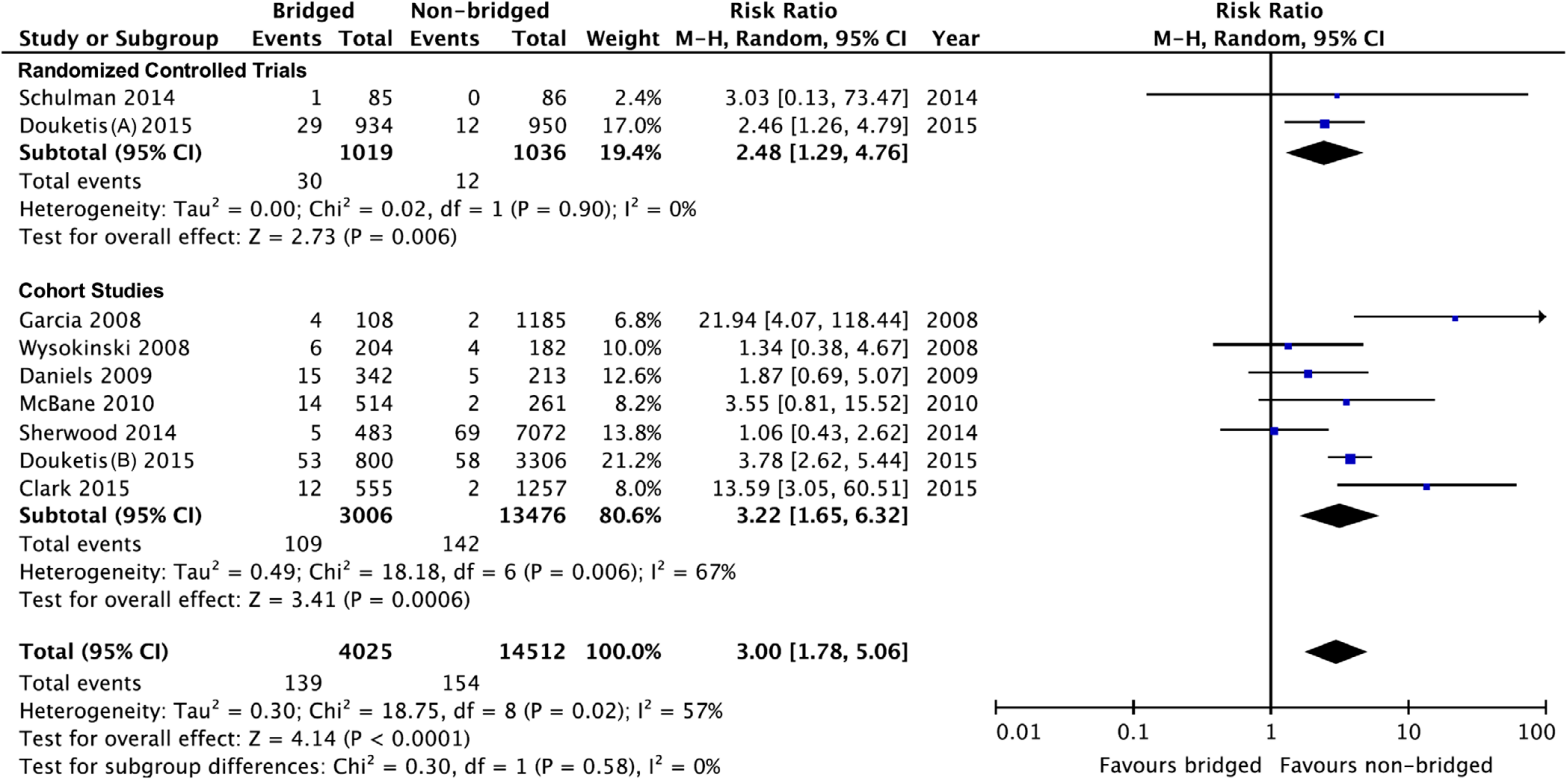
Forest plot of major bleeding between bridged and non‐bridged groups. CI, confidence interval; M‐H, Mantel‐Haenszel

The criteria for major bleeding were revealed in nine studies (two randomized trials, seven cohort studies).^10^, ^11^, ^15^, ^16^, ^22^, ^23^, ^25^, ^26^, ^27^ The analysis showed a higher risk of major bleeding associated with bridging anticoagulation compared to non‐bridging strategy with a high level of heterogeneity, RR: 3.00 (95% CI: 1.78‐5.06, *P* < .0001; *I* ^2^ = 57%; Figure 4).

### 3.4 Subgroup analysis

When comparing bridging anticoagulation with interrupted oral anticoagulant without bridging therapy, the sub‐analysis showed no significant difference in the thromboembolic risk between the two groups, RR: 1.26 (95% CI: 0.49‐3.24, *P* = .63; *I* ^2^ = 64%; Figure S1). Bridging anticoagulation was associated with an increased risk of overall bleeding compared to interrupted oral anticoagulant without bridging therapy, RR: 4.37 (95% CI: 2.64‐7.22, *P* < .0001; *I* ^2^ = 77%; Figure S2).

Similarly, when comparing bridging anticoagulation with continuous oral anticoagulation, there was no difference in the occurrence of thromboembolic events between groups, RR: 0.89 (95% CI: 0.20‐4.05, *P* = .88; *I* ^2^ = 0%; Figure S3). Bridging anticoagulation was associated with a significantly higher risk of overall bleeding compared to continuous oral anticoagulation, RR: 2.48 (95% CI: 1.28‐4.80, *P* = .007; *I* ^2^ = 62%; Figure S4).

## 4 DISCUSSION

Our analyses showed bridging anticoagulation was associated with a nearly threefold increased risk of overall and major bleeding compared to non‐bridging management and there was no significant difference in the risk of thromboembolism between the two strategies. Findings were broadly consistent across subgroups irrespective of study design.

In this study, we critically selected relevant studies and conducted a rigorously scientific synthesis of research results. Prior meta‐analyses evaluating the risk and benefit of bridging anticoagulation are flawed with inclusions of few randomized trials^6^ and studies with no control arm,^6^ no separate analysis of three strategies (interrupted anticoagulation with bridging therapy, interrupted anticoagulation without bridging therapy and continuous oral anticoagulation),^6^, ^28^ and no detailed assessment of study quality.^28^ We found that bridging anticoagulation was associated with higher bleeding risk and similar thromboembolic risk compared to non‐bridging strategy. Our subgroup analyses further showed the higher bleeding risk of bridging therapy seemed to be augmented in patients receiving interrupted anticoagulation without bridging therapy compared to those with continuous anticoagulation.

Our analysis demonstrated the risk of periprocedural thromboembolic events was 0.89% and 0.46% for the bridged and non‐bridged patients, respectively, and the difference did not reach statistical significance. Some possible reasons may explain this finding. First, most patients were classified as having a moderate risk of thromboembolism in individual studies; however, the risk‐stratification criteria were inconsistent across studies, which may particularly bias the results of observational studies, for example, indication bias (patients in a higher thromboembolic risk may be given a bridging anticoagulation). Second, the overall number of thromboembolic events was small, which may yield underpowered statistics. Third, interruption and reinitiation of warfarin may deplete protein C and protein S and thereafter contribute to a hyper‐coagulable status. Protein C and S are two vitamin K‐dependent plasma proteins that work together as a natural anticoagulant system, and deficiency in proteins C and S is associated with thrombotic tendency.^29^ Fourth, it takes at least 5 days for the international normalized ratio to normalize after stopping warfarin^29^ and NOACs are eliminated in 48 to 72 hours after discontinuation^30^; therefore, in patients who discontinued warfarin for <5 days or NOACs <48 hours, the risk of thromboembolism may remain low due to residual anticoagulation.

Although thromboembolism may cause severe morbidity and mortality, it should be weighed against the bleeding risk of bridging anticoagulation.^31^ Considering the higher risk of bleeding related to bridging anticoagulation, our results suggested non‐bridging management seems to have a favorable risk‐benefit profile in terms of thromboembolic and bleeding complications. There are some explanations for the bleeding risk of bridging anticoagulation. First, residual anti‐Xa effect or heparin‐induced thrombocytopenia may contribute to postoperative bleeding.^32^ Second, due to the interindividual variability in the sensitivity of aPTT test (the most common laboratory measurement to monitor UFH), control of aPTT range may not correlate well with the activity of bridging anticoagulation.^33^

NOACs have been increasingly used for the prevention of thromboembolic events in patients with moderate‐to‐high risk.^32^ NOACs are non‐inferior for prevention of stroke in patients with atrial fibrillation and associated with less bleeding compared to warfarin.^34^ NOACs have advantages of short half‐lives, fast onset of action, predictable pharmacokinetic properties (concentration‐dependent), and few drug‐drug interactions.^35^ Although the experience of periprocedural use of NOACs is accumulating, there are limited data available pertaining to the bridging anticoagulation for patients on NOAC therapy in terms of perioperative bleeding risk.^36^ The Dresden NOAC registry revealed major bleeding rate of 1.2% and clinically relevant nonmajor bleeding rate of 3.4% in patients using NOACs during invasive procedures.^37^ A subgroup analysis of the RE‐LY trial showed no significant difference in the risk of periprocedural major bleeding between patients using dabigatran 110 mg (3.8%), dabigatran 150 mg (5.1%), or warfarin (4.6%).^38^ However, these studies did not compare the bleeding risk between bridging and non‐bridging therapy.^37^, ^38^ Whether it is better for patients using NOACs to receive bridging anticoagulation is currently unclear. Two specific reversal agents for NOACs have been approved in the United States: idarucizumab for dabigatran reversal and andexanet alfa for apixaban and rivaroxaban reversal.^39^, ^40^ Tailoring periprocedural management of NOACs to the type of invasive procedure may reduce the risk of bleeding.

Attention to some limitations of this study is needed. First, 12 out of the 18 studies were observational, and the number of patients enrolled in the randomized controlled trials was relatively small. Second, the heterogeneity of results among studies was high, which may relate to the variations in the preoperative thromboembolic risks, types of procedure and definitions of outcomes. Third, few patients were classified as high thromboembolic risk,^13^, ^20^, ^22^, ^25^, ^26^ and the safety and benefit of bridging anticoagulation among these patients remain uncertain. Fourth, most of the included studies were relevant to warfarin‐treated patients; therefore, the results cannot be generalized to patients receiving NOACs. Fifth, types and doses of bridging regimens (UFH or LWMH; prophylactic dose or therapeutic dose), and the timing of periprocedural initiation of bridging were different across studies. Finally, we are unable to conduct subgroup analyses of high‐risk vs low‐risk procedures and outcomes of fatal bleeding due to unavailability of individual data of the included studies.

In conclusion, bridging anticoagulation was associated with increased bleeding risk compared to non‐bridging management. Besides, thromboembolism risk was similar between these two strategies. Our results do not support the use of routine bridging during the periprocedural interruption of oral anticoagulation.

## CONFLICT OF INTEREST

The authors declare no potential conflict of interest.

## AUTHOR CONTRIBUTIONS

H.C.K contributed to data acquisition, formal analysis, and manuscript writing. F.L.L. contributed to data acquisition, statistical consultation, and review. J.T.C., Y.G.C., and K.W.T. contributed to manuscript revision. Y.H.T. contributed to data verification and manuscript revision.

## Supporting information


**Figure S1** Forest plot of thromboembolic events between the bridged and the interrupted anticoagulation without bridging therapy. CI, confidence interval; M‐H, Mantel‐Haenszel.

**Figure S2** Forest plot of overall bleeding events between the bridged and the interrupted anticoagulation without bridging therapy. CI, confidence interval; M‐H, Mantel‐Haenszel.

**Figure S3** Forest plot of thromboembolic events between bridged and continued groups. CI, confidence interval; M‐H, Mantel‐Haenszel.

**Figure S4** Forest plot of overall bleeding events between bridged and continued groups. CI, confidence interval; M‐H, Mantel‐Haenszel.

**Table S1** Perioperative Bridging Strategies

**Table S2** Study Quality Assessment
