# Supplementary figures and images for: Thromboembolic and bleeding risk of periprocedural bridging anticoagulation: A systematic review and meta‐analysis
Source: Clin Cardiol. 2020 Jan 16;43(5):441–9. doi: 10.1002/clc.23336 (PMC7244304; doi:10.1002/clc.23336)

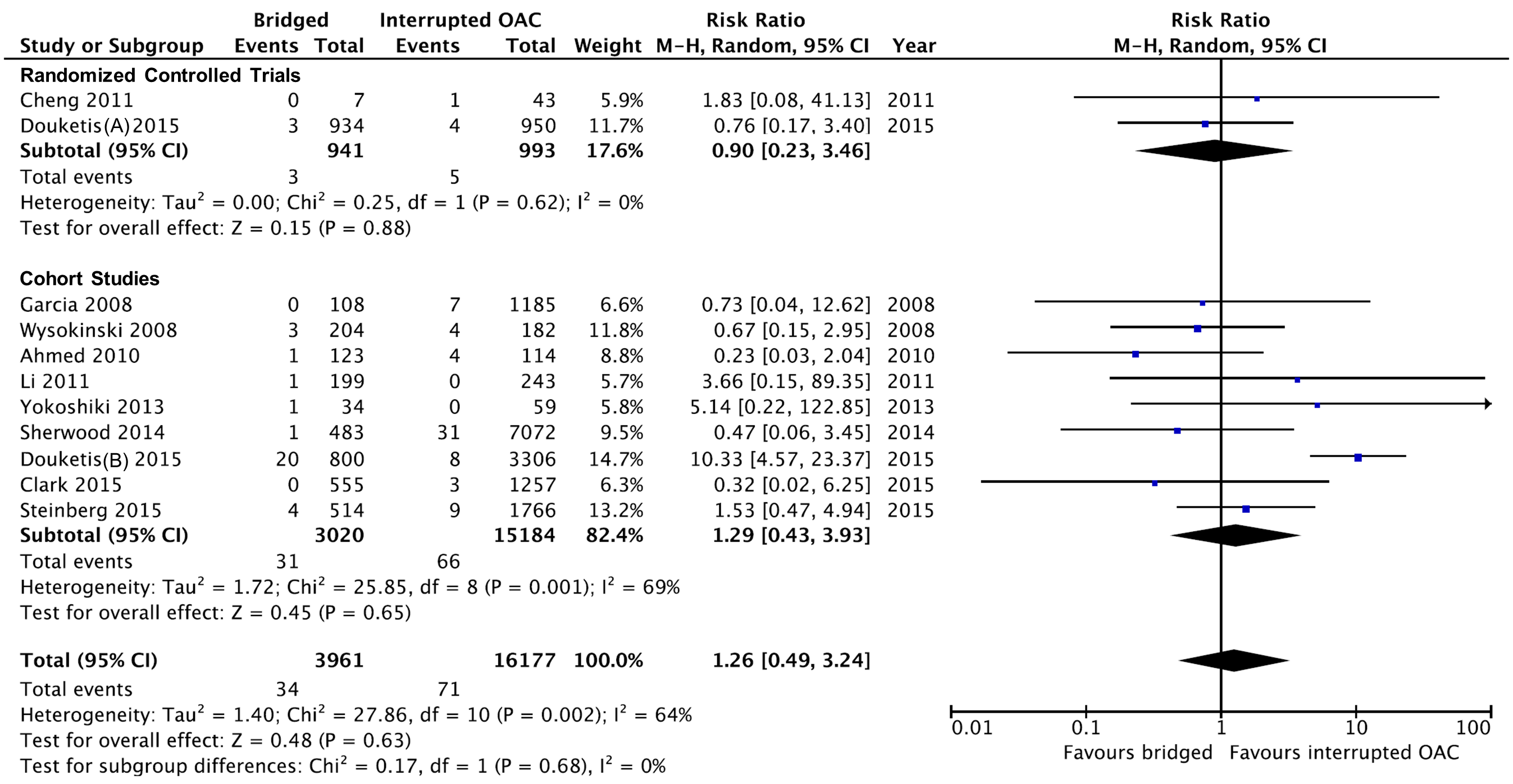

Supplement: Supplementary file 1 — Figure S1 Forest plot of thromboembolic events between the bridged and the interrupted anticoagulation without bridging therapy. CI, confidence interval; M‐H, Mantel‐Haenszel. [file CLC-43-441-s001.tif]

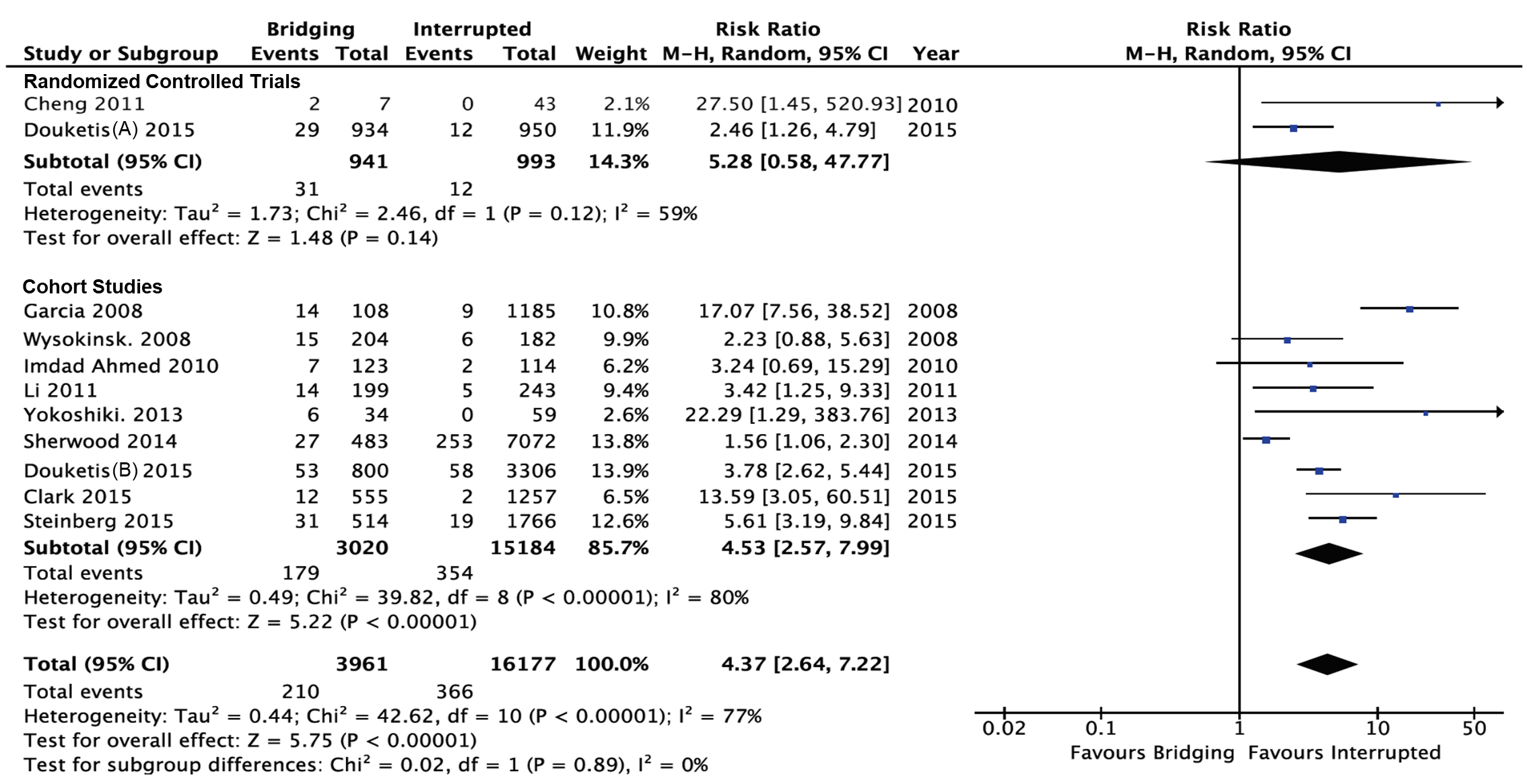

Supplement: Supplementary file 2 — Figure S2 Forest plot of overall bleeding events between the bridged and the interrupted anticoagulation without bridging therapy. CI, confidence interval; M‐H, Mantel‐Haenszel. [file CLC-43-441-s002.tif]

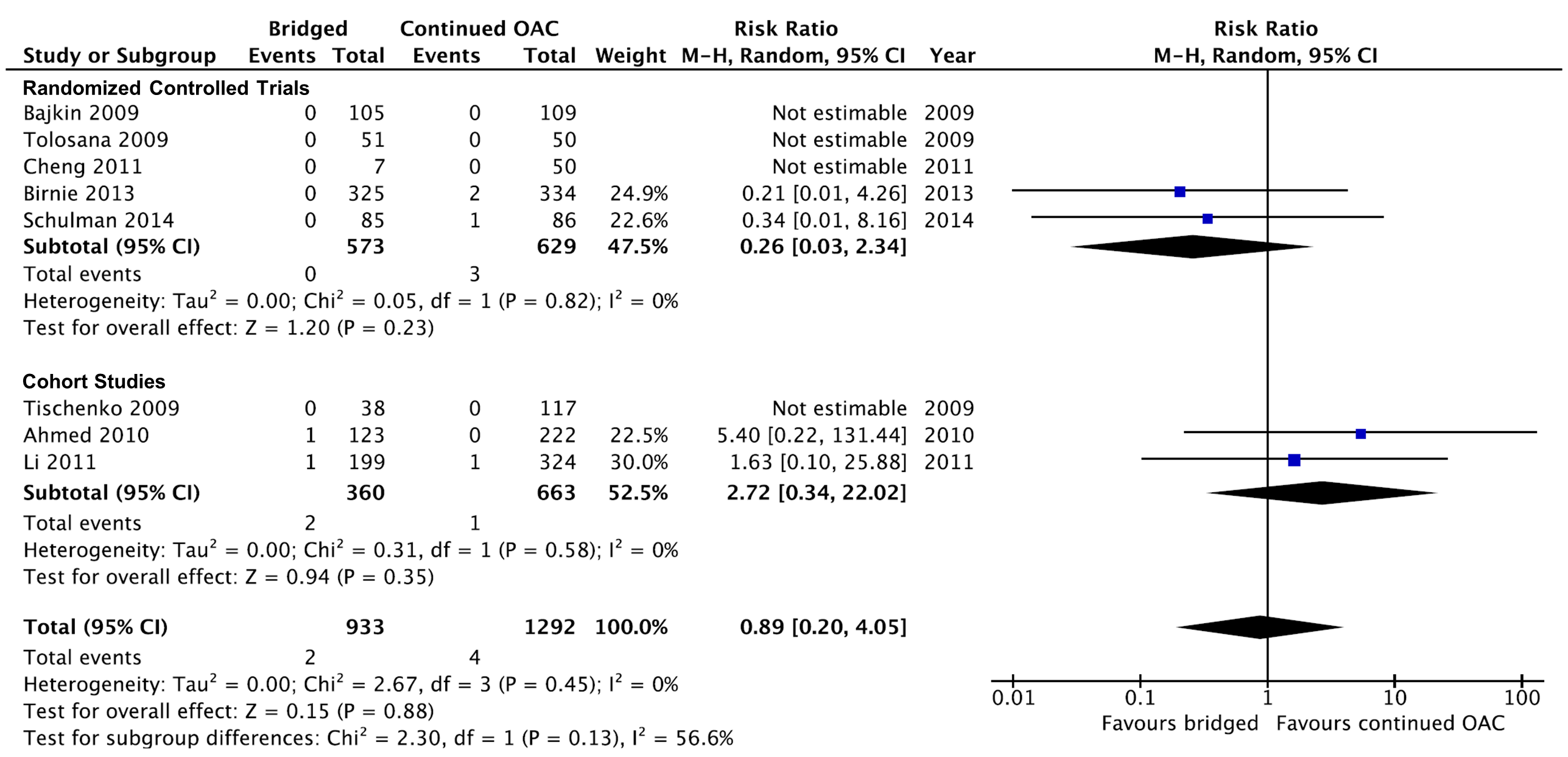

Supplement: Supplementary file 3 — Figure S3 Forest plot of thromboembolic events between bridged and continued groups. CI, confidence interval; M‐H, Mantel‐Haenszel. [file CLC-43-441-s003.tif]

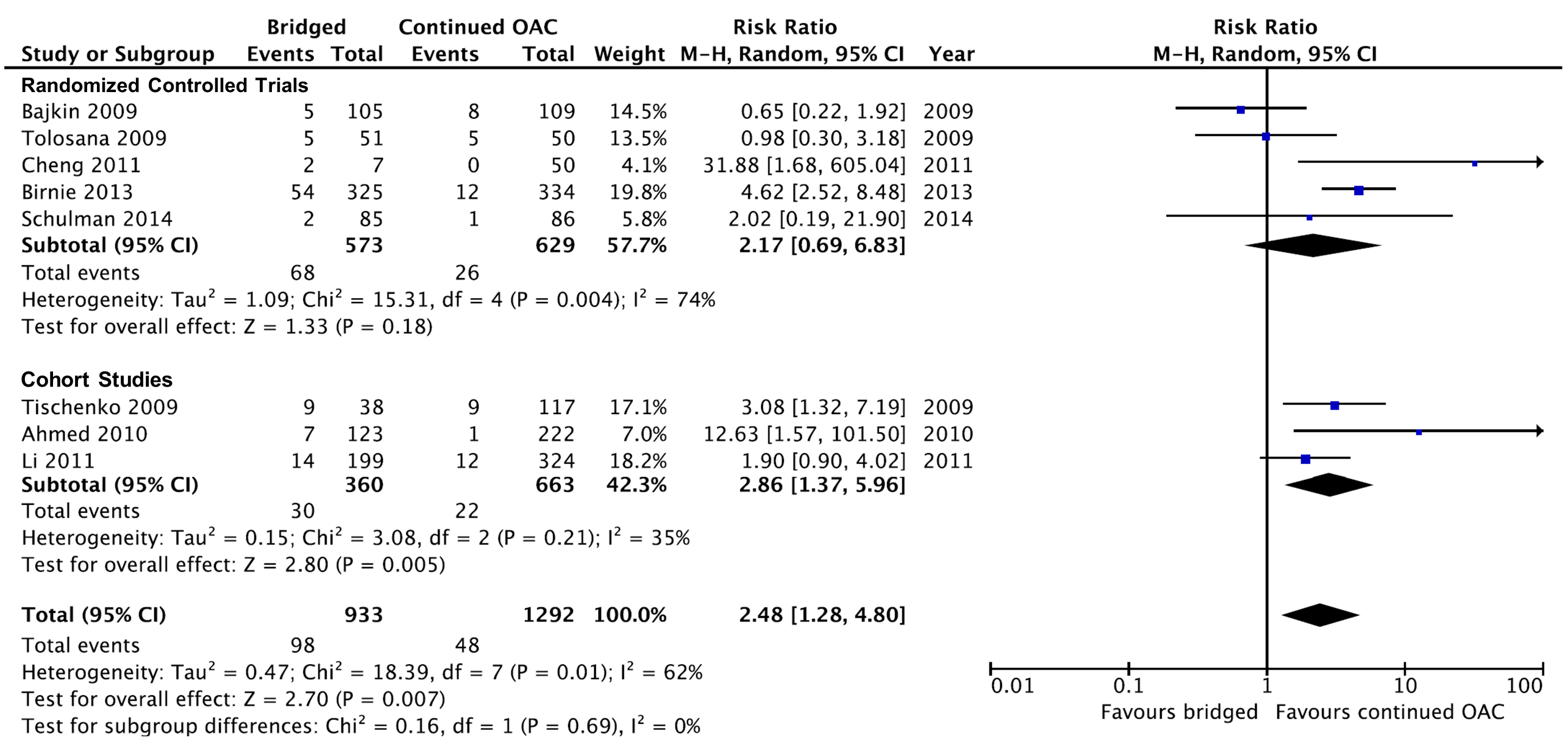

Supplement: Supplementary file 4 — Figure S4 Forest plot of overall bleeding events between bridged and continued groups. CI, confidence interval; M‐H, Mantel‐Haenszel. [file CLC-43-441-s004.tif]
