# Supplementary material for: Thromboembolic and bleeding risk of periprocedural bridging anticoagulation: A systematic review and meta‐analysis
Source: Clin Cardiol. 2020 Jan 16;43(5):441–9. doi: 10.1002/clc.23336 (PMC7244304; doi:10.1002/clc.23336)
Supplement: Supplementary file 5 — Table S1 Perioperative Bridging Strategies [file CLC-43-441-s005.docx]

**Supplementary Table S1. Perioperative Bridging Strategies**

|  | Studies, n (%) |
| --- | --- |
| Preoperative strategy |  |
| VKA discontinuation, day (18 studies)* |  |
| < 3 | 0 (0) |
| ≥ 3 | 13 (72%) |
| Unspecified or varied | 5 (28%) |
| Type of heparin bridging (18 studies) |  |
| LMWH | 7 (39%) |
| UFH | 2 (11%) |
| LMWH or UFH | 9 (50%) |
| LMWH discontinuation, hour (16 studies) |  |
| < 24 | 3 (19%) |
| ≥ 24 | 7 (44%) |
| Unspecified | 6 (37%) |
| Postoperative strategy |  |
| Reinitiation of VKA, hour (18 studies)* |  |
| < 24 | 10 (56%) |
| ≥ 24 | 2 (11%) |
| Unspecified or varied | 6 (33%) |
| VKA dose (18 studies) |  |
| Maintenance dose | 6 (33%) |
| Loading dose | 2 (11%) |
| Unspecified | 12 (67%) |
| Type of heparin bridging (15 studies) |  |
| LMWH | 5 (33%) |
| UFH | 2 (13%) |
| LMWH or UFH | 8 (53%) |
| LMWH reinitiation (13 studies), hour* |  |
| < 24 | 0 (0) |
| ≥ 24 | 7 (54%) |
| Unspecified or varied | 6 (46%) |

VKA, vitamin K antagonist; LMWH, low-molecular-weight heparin; UFH, intravenous unfractionated heparin.

* Individual studies may have used different strategies of VKA discontinuation period, VKA reinitiation time, and LMWH reinitiation time
