# Supplementary material for: Thromboembolic and bleeding risk of periprocedural bridging anticoagulation: A systematic review and meta‐analysis
Source: Clin Cardiol. 2020 Jan 16;43(5):441–9. doi: 10.1002/clc.23336 (PMC7244304; doi:10.1002/clc.23336)
Supplement: Supplementary file 6 — Table S2 Study Quality Assessment [file CLC-43-441-s006.docx]

**Supplementary Table S2. Study Quality Assessment**

| **Randomized Controlled Trials** | | | | | | |
| --- | --- | --- | --- | --- | --- | --- |
| **Author** | **Random Allocation** | **Allocation Concealment** | **Blinding** | **Unavailable for Follow-Up** | **Analysis** | |
| **Bajkin et al, 2009 ^12^** | PY | PN | Patients, CN; caregivers, CN; data collectors, NR adjudicators, NR; data analysis, NR | NR | ITT: NR for efficacy outcomes  Data for primary efficacy assessment available for 100.0% of randomized patients | |
| **Tolosana et al, 2009 ^14^** | DY | DY | Patients, CN; caregivers, CN; data collectors, DY adjudicators, DY; data analysis, DY | NR | ITT: DY for efficacy outcomes  Data for primary efficacy assessment available for 100.0% of randomized patients | |
| **Cheng et al, 2011 ^18^** | DY | PN | Patients, CN; caregivers, CN; data collectors, NR adjudicators, PY; data analysis, NR | NR | ITT: DY for efficacy outcomes  Data for primary efficacy assessment available for 96.2% of randomized patients | |
| **Birnie et al, 2013 ^20^** | DY | DY | Patients, CN; caregivers, CN; data collectors, NR adjudicators, DY; data analysis, NR | DY | ITT: DY for efficacy outcomes  Data for primary efficacy assessment available for 96.8% of randomized patients | |
| **Schulman et al, 2014 ^23^** | DY | PN | Patients, CN; caregivers, CN; data collectors, NR adjudicators, NR; data analysis, NR | DY | ITT: PN for efficacy outcomes  Data for primary efficacy assessment available for 99.4% of randomized patients | |
| **Douketis (A) et al, 2015 ^25^** | DY | DY | Patients, DY; caregivers, DY; data collectors, NR adjudicators, DY; data analysis, DY | DY | ITT: PY for efficacy outcomes  Data for primary efficacy assessment available for 95.8% of randomized patients | |
| **Observational Studies** | | | | | | |
| **Author** | **Consecutive Enrollment** | **Protocol in Place Before Enrollment** | **Intervention/Control Setting Similar** | **Intervention/Control Time Frame Similar** | **Blinded Assessment of Outcome** | **Loss to Follow-Up Reported** |
| **Wysokinski et al, 2008 ^10^** | Yes | No | Yes | Yes | Yes | Yes |
| **Garcia et al, 2008 ^11^** | No | No | No | Yes | No | Yes |
| **Tischenko et al, 2009 ^13^** | Yes | Yes | Yes | No | No | No |
| **Daniels et al, 2009 ^15^** | Yes | Yes | Yes | Yes | Yes | Yes |
| **McBane et al, 2010 ^16^** | Yes | Yes | Yes | Yes | Yes | Yes |
| **Ahmed et al, 2010 ^17^** | Yes | Yes | Yes | Yes | No | No |
| **Li et al, 2011 ^19^** | No | No | Yes | Yes | No | No |
| **Yokoshiki et al, 2013 ^21^** | Yes | No | Yes | No | No | No |
| **Sherwood et al, 2014 ^22^** | Yes | Yes | Yes | Yes | No | Yes |
| **Steinberg et al, 2015 ^24^** | Yes | No | No | Yes | No | Yes |
| **Douketis (B) et al, 2015 ^26^** | Yes | Yes | Yes | Yes | Yes | Yes |
| **Clark et al, 2015 ^27^** | Yes | No | Yes | Yes | No | No |

DY, definitely yes; PY, probably yes; PN, probably no; CN, certainly no; NR, not reported; ITT, intent to treat.
